# Integrative Analysis for Identifying Co-Modules of Microbe-Disease Data by Matrix Tri-Factorization With Phylogenetic Information

**DOI:** 10.3389/fgene.2020.00083

**Published:** 2020-02-21

**Authors:** Yuanyuan Ma, Guoying Liu, Yingjun Ma, Qianjun Chen

**Affiliations:** ^1^ School of Computer and Information Engineering, Anyang Normal University, Anyang, China; ^2^ School of Computer, Central China Normal University, Wuhan, China; ^3^ School of Life Science, Hubei University, Wuhan, China

**Keywords:** microbe-disease association, matrix factorization, phylogenetic distance, human microbiome, co-modules

## Abstract

Microbe-disease association relationship mining is drawing more and more attention due to its potential in capturing disease-related microbes. Hence, it is essential to develop new tools or algorithms to study the complex pathogenic mechanism of microbe-related diseases. However, previous research studies mainly focused on the paradigm of “one disease, one microbe,” rarely investigated the cooperation and associations between microbes, diseases or microbe-disease co-modules from system level. In this study, we propose a novel two-level module identifying algorithm (MDNMF) based on nonnegative matrix tri-factorization which integrates two similarity matrices (disease and microbe similarity matrices) and one microbe-disease association matrix into the objective of MDNMF. MDNMF can identify the modules from different levels and reveal the connections between these modules. In order to improve the efficiency and effectiveness of MDNMF, we also introduce human symptoms-disease network and microbial phylogenetic distance into this model. Furthermore, we applied it to HMDAD dataset and compared it with two NMF-based methods to demonstrate its effectiveness. The experimental results show that MDNMF can obtain better performance in terms of enrichment index (EI) and the number of significantly enriched taxon sets. This demonstrates the potential of MDNMF in capturing microbial modules that have significantly biological function implications.

## Introduction

With the development of high-throughput sequencing technology, such as 16S ribosomal RNA (16S rRNA), more and more microbes were identified. Nearly 10^14^ bacterial cells are existed in human internal gut and provide a wide variety of gene products which induce diverse metabolic activities (Micah et al., 2007; Shah et al., 2016). The dynamic balance of human microbiome composition is essential to maintain good health. Once such balance is broken, many closely related human disease and disorders may be caused (Medzhitov, 2007; Thiele et al., 2013), such as colorectal cancer (CRC) (Boleij et al., 2014), obesity (Turnbaugh et al., 2009), inflammatory bowel disease (IBD) (Qin et al., 2010), bacterial vaginosis (Fredricks et al., 2005), and so on. For example, Jorth et al. have reported that gene expression profiles of periodontitis-related microbial communities have highly conserved changes, relative to healthy samples (Jorth et al., 2014). It means that microbiome composition changes in oral cavity could be associated with pathogenesis of periodontitis. Furthermore, Socransky et al. have found that subgingival plaque is connected with several major microbial taxon including *Fusobacterium*, *Prevotella*, and so on (Socransky et al., 1998). Chen et al. have observed that the colonization with *Helicobacter pylori* has negative correlation with the symptom of allergy (pollens and molds), especially in the childhood (Chen and Blaser, 2007; Blaser, 2014). All these reveal the potential association between pathogenic microorganisms and complex human diseases.

Considering the key role of microbes in health, many important projects including the Human Microbiome Plan (HMP) (Gevers et al., 2012), the Earth Microbiome Project (EMP) (Gilbert et al., 2010), Metagenomics of the Human Intestinal Tract (MetaHIT) (Ehrlich and Consortium, 2011) were launched to investigate the relationships between microbiota and diseases. Moreover, some related databases and tools have been developed to analyze the increasing information for disease-related microbes. A human microbe-disease association database, called HMDAD (Ma et al., 2016a), manually collected 483 microbe-disease association entries from previously published literatures. These databases provide a possibility for microbe-disease association relationship prediction by computational approaches. Zhang et al. proposed bidirection similarity integration method (BDSILP) for predicting microbe-disease associations by integrating the disease-disease semantic similarity and the microbe-microbe functional similarity. Wang et al. proposed a semisupervised computational model called LRLSHMDA to predict large-scale microbe-disease association (Wang et al., 2017). Huang et al. combined neighbor-based collaborative filtering and graph-based model into a unified objective function to predict microbe-disease relationship (Huang et al., 2017). He et al. integrated symptom-based disease similarity network into graph regularized nonnegative matrix factorization models (GRNMF), meanwhile utilizing neighbor information to boost the performance of GRNMF (He et al., 2018). Zhang et al. utilized the advantages of ensemble learning to improve the performance of association prediction, which provided a new way for mining microbe-disease relationship (Zhang et al., 2018a; Zhang et al., 2019). All these efforts pave the way for further understanding complex regulatory mechanisms by means of which disease-related microbiota get involved.

However, cellular system is complicatedly organized and biological functions are mainly performed in a highly modular manner (Barabasi and Oltvai, 2004; Chen and Zhang, 2018). In microbial ecosystems, microbes often cooperate with each other to finish some biochemical activities. For example, *ammonifiers* decompose nitrogen-containing organic compounds to release ammonia. *Nitrous acid bacteria* (also known as *ammonia oxidizing bacteria*) oxidize ammonia to nitrous acid. Then, *nitric acid bacteria* (also known as *nitrous acid oxidizing bacteria*) oxidize nitrous acid to nitric acid. These two types of bacteria can obtain the energy needed for growth from the above oxidation process. Therefore, the mutualism relationship among *ammonifier*, *nitrous acid bacteria*, and *nitric acid bacteria* forces them to form a tight biological community. Guo et al. studied the contributions of high-order metabolic interactions to the activity of four-species microbial community and demonstrated that the interactions between pairwise species play an important role in predicting the complex cellular network behavior (Guo and Boedicker, 2016). Although knowledge about microbe-disease associations could provide helpful insights into understanding complex disease mechanisms (Huang et al., 2017; He et al., 2018), the “one-disease, many microbes” models ignore interactions within microbial community composed of several species.

Recently, multilayer interaction and modular organization have attracted more and more attentions. Several studies proposed co-module discovery methods to identify combinatorial patterns using pairwise gene expression and drug response data (Kutalik et al., 2008; Chen and Zhang, 2016). In addition, Chen et al. proposed a new method based nonnegative matrix factorization (NMF) to reveal drug-gene module connections from different molecular levels (Chen and Zhang, 2018). Cai et al. proposed a new network-guided sparse binary matching model to jointly analyze the gene-drug patterns hidden in the pharmacological and genomic datasets with the additional prior information of genes and drugs (Cai et al., 2018). Chen et al. also proposed a higher order graph matching with multiple network constraints (gene network and drug network) to identify co-modules from different multiple data sources (Chen et al., 2018).

All these have made great progresses to study the coordinate regulatory mechanisms between two or more biological molecular networks from a systematic view. However, as far as we know, less work focuses on microbe-disease co-modules discovering. Previous studies mainly aimed to microbe-disease association prediction, and did not reveal within-module interactions (microbe-microbe, disease-disease) from the same level and cross-module interactions (microbe-disease) from multiple molecular levels.

**Figure 1:**
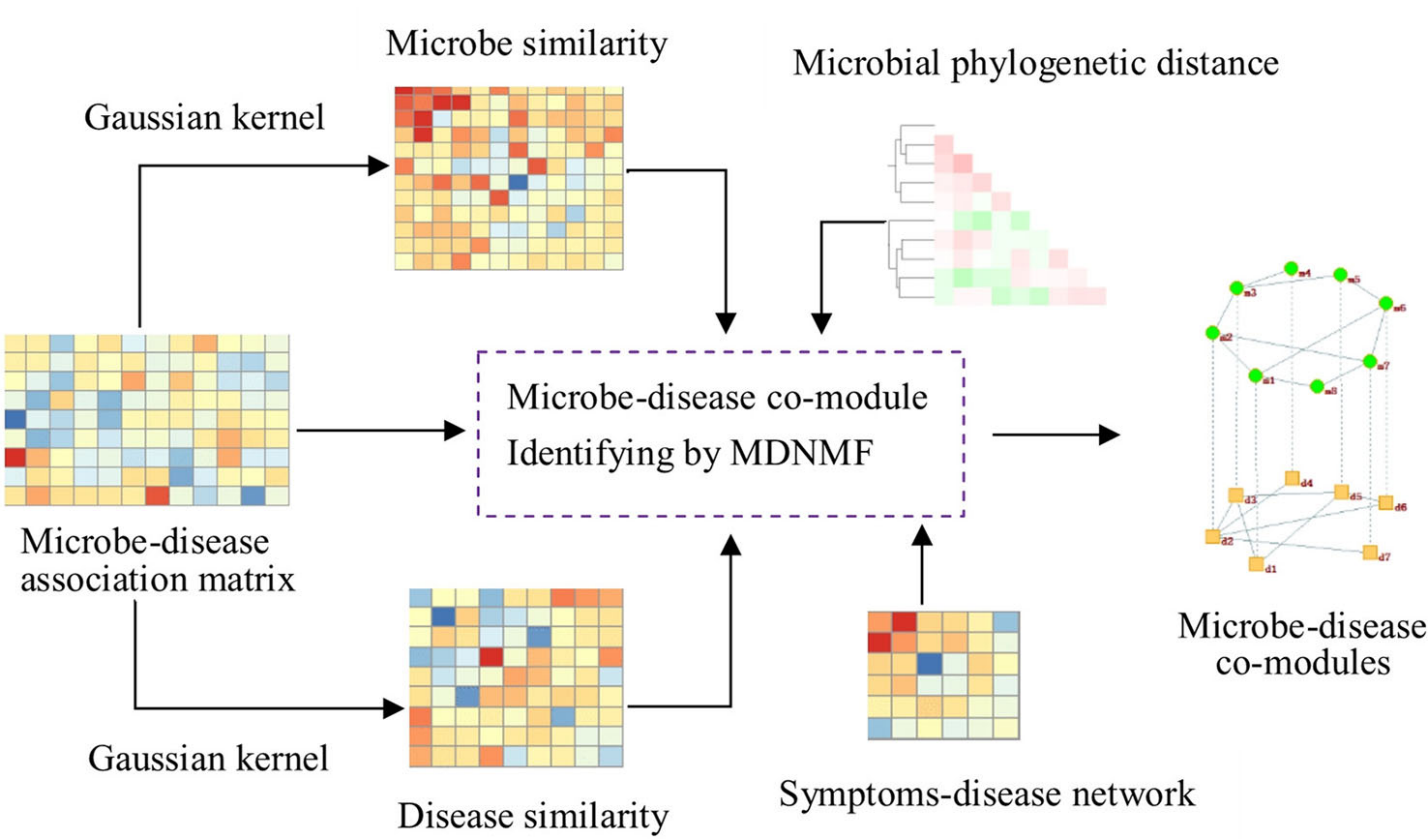
Illustrative example of MDNMF. First, based on Gaussian kernel function we can obtain microbe and disease similarity matrices from the original microbe-disease association matrix. Then, these three matrices are served as the input of MDNMF. Simultaneously, in order to improve the accuracy of module finding and biological interpretability of modules identified by MDNMF, human symptoms-disease network and microbial phylogenetic distance are also introduced into the model. At last, microbe-disease co-modules from different levels can be obtained.

To this end, we design a new algorithm based on NMF to construct the two-level microbe-disease module network by Gaussian profile kernel similarity (MDNMF). In order to improve efficiency and effectiveness of the proposed algorithm, we introduce human symptoms-disease network (Zhou et al., 2014) and microbial phylogenetic distance into this model, which makes functionally similar microbes (diseases with similar symptoms) tend to appear in the same microbial module (disease module). We applied MDNMF to HMDAD dataset and compared it with two classical NMF methods to demonstrate its effectiveness. The experimental results show that the majority of identified microbial modules have significant functional implications [significantly enriched in taxon sets that refer to groups of microbes that has something in common (Dhariwal et al., 2017)]. **Figure 1** gives the illustrative example of MDNMF.

The contribution of this paper lies in (1) an efficient two-level module discovering algorithm (MDNMF) has been proposed to reveal microbe-microbe, disease-disease and microbe-disease modules association. (2) The phylogenetic distance of disease-related microbes is introduced into the proposed MDNMF model to make phylogenetically close microbes tend to intertwine in the development of similar disease. To our knowledge, this is the first attempt to link microbial phylogenetic relatedness to NMF-based module identification. (3) The proposed MDNMF algorithm is easily extended to other multiple-level molecular network application, for example, virus-host co-modules, microbe-drug co-modules discovering, and so on. The rest of this paper is organized as: in the next section, we give a brief overview of NMF and MDNMF. And then, followed by the experimental results and the conclusions are provided in the last section.

## Materials and Methods

### Dataset

The dataset is downloaded from the Human Microbe-Disease Association Database (HMDAD, http://www.cuilab.cn/hmdad) (Ma et al., 2016a). It contains 483 microbe-disease associations, which cover 292 microbes and 39 diseases. By 16S RNA sequencing techniques, most microbe names was recorded at the genus level. Based on these known microbe-disease relation, an adjacency matrix *X*∈ℝ^292×39^ can be constructed where *X_ij_*=1 if microbe *i* is related to disease *j*, and vice versa.

### The NMF Model

NMF and its variants have been widely applied to various fields including bioinformatics (Ma et al., 2016b; Ma et al., 2017; Chen and Zhang, 2018). In NMF, given an original data matrix *X*∈ℝ*^n^* ^×^ *^m^*, we seek to find two low-rank matrices *W*∈ℝ*^n^* ^×^ *^k^* (also called basis matrix) and *H*∈ℝ*^k^* ^×^ *^m^* (coefficient matrix) to approximate *X*, such that *X*≈*WH*, where *k*<<min(*m*,*n*). Here, data *X* can be represented as the linear additional combination of basis vectors. We can obtain such a decomposition by solving the following least squares problem:

(1) $$\underset{W,H\geq 0}{\min}{\left\|X-WH\right\|}_{F}^{2},$$

where ||•||*_F_* denotes Frobenius norm.

### Gaussian Interaction Profile Kernel Similarity for Microbes

Based on the hypothesis that functionally similar microbes could be associated with more common human diseases, Gaussian kernel interaction profiles can be used to calculate the inferred microbe similarity (Wang et al., 2017; He et al., 2018). Given microbe-disease association matrix *X*, the *ith* row of *X* indicates the interaction profiles between microbe *m_i_* and all the diseases. For any two microbes *m_i_* and *m_j_*, their similarity can be computed as follows:

(2) $$MS(m_{i},m_{j})=\exp\left(-\gamma_{m}{\left\|X_{i,}*-X_{j,}*\right\|}^{2}\right),$$

where X_i,*_ denotes the *ith* row of matrix *X*. γ*_m_* is bandwidth parameter that needs to be normalized based on a novel bandwidth parameter γ′*_m_* and the interaction profile for each microbe, i.e., the *ith* row of *X*:

(3) $$\gamma_{m}=\gamma_{m}^{\prime }/\left(\frac{1}{n_{m}}\;\sum_{i=1}^{n_{m}}{\left\|X_{i,}*\right\|}^{2}\right).$$

Here, *n_m_* is the number of microbes related to all diseases (here, *n_m_*=292). γ′*_m_* was set as 1 according to the previous study (Wang et al., 2017). In this way, microbe similarity matrix *MS* can be constructed, the element of *MS* indicates the similarity score between two arbitrary microbes.

### Gaussian Interaction Profile Kernel Similarity for Diseases

Similarly, Gaussian kernel based disease similarity matrix can be inferred as follows:

(4) $$DS(d_{i},d_{j})=\exp\left(-\gamma_{d}{\left\|{X*}_{,i}-{X*}_{,j}\right\|}^{2}\right)$$

(5) $$\gamma_{d}=\gamma_{d}^{\prime }/\left(\frac{1}{n_{d}}\sum_{i=1}^{n_{d}}{\left\|{X*}_{,i}\right\|}^{2}\right),$$

where *X* _*,i_ denotes the *ith* column of *X*, *n_d_* is the number of diseases related to all microbes (*n_d_*=39), γ′*_d_* was also assigned to 1.

### Phylogenetic Distance for Disease-Related Microbes

Gaussian interaction profiles kernel similarity reflects the intertwining between microbes in term of microbe-disease association relationship. However, functionally similarity could not be explained only by disease relatedness, homology and phylogenetic correlation should be considered as side information to make the connected microbes in the microbe-disease association matrix likely to be placed in the same co-modules.

We searched 91 nucleotide sequences of disease-related microbes from NCBI, and imported them into MEGA to compute the phylogenetic distance between pairwise sequences by Kimura 2-parameter model. Other parameters are set in default. Thus, we can obtain the final microbial phylogenetic distance matrix *M _phy_* which is used to enforce microbe members within identified modules likely to be near in phylogeny.

In order to demonstrate the role of phylogenetic information in identifying disease-related microbe modules, we extract the top 10 largest and smallest phylogenetic distance pairs as illustrative examples to further analyze whether closely related taxa tend to associate with the same disease, or similar diseases. For each microbe-microbe phylogenetic distance pair, we compute the Jaccard coefficient (JC) between two microbe-related disease profiles (rows of microbe-disease association matrix). The results shows that top 10 microbe pairs which are closely related in genetic have the largest JCs in terms of disease profile similarities. Similarly, we also compute the disease similarities between phylogenetically distant microbes and find that 9 in 10 microbe pairs have the smallest JCs. This suggests that closely related taxa tend to associate with the same disease or similar diseases, and phylogenetically distant taxa usually have distinct disease profiles.

### The MDNMF Algorithm

Besides the typical NMF as *Dataset* described, tri-factor NMF (tri-NMF, *X*≈*FSG*) is also an important matrix factorization method for clustering (Ding et al., 2006). In tri-NMF, factorized matrices *F*,*G* provide an approach to perform biclustering of *X*, respectively. Factorized matrix *s* not only provides an additional degree of freedom to enforce the reconstruct error tiny, but also implicitly denotes the relationship between clusters (Ding et al., 2005). In particular, given the symmetric similarity matrix *A*, we can decompose it into *A*≈*HS_H_^T^*. The similarity matrix reflects the intrinsic connection patterns within its original data matrix (Van Dam et al., 2017). In this paper, we propose a novel algorithm MDNMF to simultaneously factorize two similarity matrices (microbe similarity matrix *MS*, disease similarity matrix *DS*) and one microbe-disease association matrix *X*. The objective function is formulated as follows:

(6) $$\begin{array}{c} \underset{H_{1},{\;H}_{2},{\text{ S}}_{1},\;{\text{S}}_{2}}{\min}\;{\left\|MS-H_{1}S_{1}H_{1}^{T}\right\|}_{F}^{2}+\lambda_{1}{\left\|X-H_{1}H_{2}^{T}\right\|}_{F}^{2}+\lambda_{2}{\left\|DS-H_{2}S_{2}H_{2}^{T}\right\|}_{F}^{2}\\ \text{s}.\text{t}.\;H_{1},\;H_{2},\;S_{1},\;S_{2}\geq 0. \end{array}$$

where *MS* ∈ℝ*^n^m* ^×^ *^n^m*, *DS*∈ℝ*^n^d* ^×^ *^n^d* are microbe-microbe and disease-disease similarity matrices, respectively. *H*_1_∈ℝ*^n^m* ^×^ *^k^*, *H*_2_∈ℝ*^n^d* ^×^ *^k^* are cluster indication matrices, *S*_1_∈ℝ*^k^* ^×^ *^k^*, *S*_2_∈ℝ*^k^* ^×^ *^k^* are the symmetric matrices. Here, *k* is the number of clusters, and λ_1_, λ_2_ are the parameters to balance the weights of three terms in Eq.6. The second term${\left\|X-H_{1}H_{2}^{T}\right\|}_{F}^{2}$ establishes the one-to-one relationships between identified microbe modules and disease modules. Moreover, it can be regarded as a tri-NMF${\left\|X-H_{1}{IH}_{2}^{T}\right\|}_{F}^{2}$, here *I* is the identity matrix which enforce the *ith* module identified by microbe clustering indication matrix *H*_1_ is only bound up with the *ith* module by *H*_2_. The other two terms respectively identify one type of modules at individual levels and reveal the module associations within them *via S*_1_ and *S*_2_.

In order to further improve the performance of the proposed algorithm, we introduce symptoms-based disease similarity network and microbial phylogenetic distance into MDNMF. The symptoms-based disease similarity was previously studied based on co-occurrence of disease/symptom terms (Zhou et al., 2014). Here, we use *DS_sym_* to denote symptoms-based disease similarity matrix. The objective function of MDNMF (Eq.6) can be rewritten as follows:

(7) $$\begin{array}{c} \underset{H_{1},{\;H}_{2},{\text{ S}}_{1},\;{\text{S}}_{2}}{\min}\;{\left\|MS-H_{1}S_{1}H_{1}^{T}\right\|}_{F}^{2}+\lambda_{1}{\left\|X-H_{1}H_{2}^{T}\right\|}_{F}^{2}+\lambda_{2}{\left\|DS-H_{2}S_{2}H_{2}^{T}\right\|}_{F}^{2}+\mu \left(tr\left(H_{1}^{T}L_{1}H_{1}\right)+tr\left(H_{2}^{T}L_{2}H_{2}\right)\right)\\ {\text{s}.\text{t}.\;H}_{1},\;H_{2},\;S_{1},\;S_{2}\geq 0. \end{array}$$

Where *L*_1_=*D*_1_-*MS_phy_*, *L*_2_=*D*_2_-*DS_symp_* are Laplacian matrices, ${\left(D_{1}\right)}_{i}=\sum_{j}{\left({MS}_{phy}\right)}_{ij}$,${\left(D_{2}\right)}_{i}=\sum_{j}{\left({DS}_{symp}\right)}_{ij}$ are degree matrices, respectively. *MS_phy_*=1-*M_phy_μ* is the regularization parameter and the whole last term in Eq.7 is used to exert a penalty for violating the prior cognition about microbial phylogeny and disease phenotype associations.

Note that disease symptoms dataset collected from PubMed literatures contains diseases and symptoms terms. The association between symptoms and diseases are quantified using term co-occurrence (just like in the field of information retrieval, if the document and keyword simultaneously appear, the corresponding position of the word-document matrix is set to the frequency of co-occurrence). And then, each disease can be represented by a vector of symptoms. At last, the cosine similarity function is used to quantify the similarity between two diseases. The link weight between two diseases quantifies the similarity of their respective symptoms. Thus, these two disease similarities based on microbes and human symptoms are different essentially in that HMDAD dataset describes the binary relationships between microbes and diseases, however, disease symptoms dataset describes the co-occurrence relationships between symptoms and diseases. Integrating them into the objective of MDNMF will simultaneously take account of the diffusion and propagation of the information from different source.

We used the multiplicative update rules to solve MDNMF problem and can find a local minimal solution by alternately updating matrices *H*_1_, *H*_2_, *S*_1_, *S*_2_.

(1) Fix *H*_1_,*H*_2_, *S*_2_ and update *S*_1_ with

(8) $${\left(S_{1}\right)}_{ij}\leftarrow {\left(S_{1}\right)}_{ij}\frac{{\left(H_{1}^{T}MSH_{1}\right)}_{ij}}{{\left(H_{1}^{T}H_{1}S_{1}H_{1}^{T}H_{1}\right)}_{ij}}$$

(2) Fix *H*_1_, *H*_2_, *S*_1_ and update *S*_2_ with

(9) $${\left(S_{2}\right)}_{ij}\leftarrow {\left(S_{2}\right)}_{ij}\frac{{\left(H_{2}^{T}DSH_{2}\right)}_{ij}}{{\left(H_{2}^{T}H_{2}S_{2}H_{2}^{T}H_{2}\right)}_{ij}}$$

(3) Fix *S*_1_, *S*_2_, *H*_2_ and update *H*_1_ with

(10) $${\left(H_{1}\right)}_{ij}\leftarrow {\left(H_{1}\right)}_{ij}\frac{{\left(2MSH_{1}S_{1}\;+{\;\lambda }_{1}XH_{2}\text{ + }\mu D_{1}H_{1}\right)}_{ij}}{{\left({2H_{1}S_{1}H}_{1}^{T}H_{1}S_{1}+{\lambda_{1}H}_{1}H_{2}^{T}H_{2}+\mu {MS}_{phy}H_{1}\right)}_{ij}}$$

(4) Fix *S*_1_, *S*_2_, *H*_1_ and update *H*_2_ with

(11) $${\left(H_{2}\right)}_{ij}\leftarrow {\left(H_{2}\right)}_{ij}\frac{{\left(2\lambda_{2}DSH_{2}S_{2}\;+{\;\lambda }_{1}X^{T}H_{1}\text{ + }\mu D_{2}H_{2}\right)}_{ij}}{{\left(2{\lambda_{2}H}_{2}S_{2}H_{2}^{T}H_{2}S_{2}\;+\;{\lambda_{1}H}_{2}H_{1}^{T}H_{1}\;+\;\mu {DS}_{symp}H_{2}\right)}_{ij}}$$

### Determination of Modules

In fact, the same microbe may play different roles in the development of diseases. Therefore, the idea of soft clustering is more suitable to model the function associations among microbes. The factorized matrices *H*_1_, *H*_2_ can be used to identify two types of modules, respectively. The elements with relatively large values of each column of *H*_1_ (*H*_2_) is assigned to the members of corresponding module. We calculate the threshold for each feature (each row$h_{i,*}^{1}$of *H*_1_ ($h_{i,*}^{2}$of *H*_2_)) with

(12) Th(f)=μ(f)+tσ(f),

where $\mu \left(f\right)=\frac{1}{k}\sum_{k}h_{fk}$, $\sigma \left(f\right)=\sqrt{\frac{1}{n-1}\sum_{k}{\left(h_{fk}-\mu \left(f\right)\right)}^{2}}$, *t* is a given threshold. Based on this rule, we determined the *ith* module members if the entries of $h_{fi}^{*}$ are larger than *Th* (*f*). In *Experimental Results and Discussion* section, we set *t*=1.5 for two clustering indication matrices *H*_1_ and *H*_2_ to identify modules with proper resolution.

### Determination of Module Links

Given the symmetric similarity matrix *A*, tri-NMF factorizes it to be $A\approx HSH^{T}=\sum_{i=1}^{k}\sum_{j=1}^{k}s_{ij}h_{i}h_{j}^{T}$. Here, *h_i_* denotes the *ith* column of *H*, *s_ij_* is the corresponding element of s. The latent clustering indication vector *h_i_* can reconstruct the original similarity matrix *A*, and *s_ij_* can be viewed as the weight of $h_{i}h_{j}^{T}$. It means that the larger *s_ij_* is, the stronger the connection between the modules identified by *h_i_* and *h_j_* is. Therefore, the diagonal elements of *s* can be used to evaluate the quality of clustering, and the off-diagonal elements can be used to establish the possible connections between different modules.

### Functional Enrichment Analysis for Co-Modules

We use MicrobiomeAnalyst (Dhariwal et al., 2017) tools to conduct functional enrichment analysis for microbe modules, and select the significantly enriched taxon set terms if *P*-value < 0.005 and FDR < 0.05 (hypergeometric tests). Because MicrobiomeAnalyst provides 229 taxon sets associated with host-intrinsic factors such as diseases. For microbe-disease co-modules we define the enrichment indices between significantly enriched taxon set terms and diseases within the same co-module to evaluate the performance of different algorithms. The enrichment index (EI) is formulated as follows:

(13) $$EI=\frac{\left|\left\{\mathrm{significantly}\text{ enriched }\mathrm{taxon}\text{ set}\right\}\cap \left\{\mathrm{diseases}\right\}\right|}{\left|\left\{\mathrm{significantly}\text{ enriched }\mathrm{taxon}\text{ set}\right\}\cup \left\{\mathrm{diseases}\right\}\right|},$$

where |{*significantly* enriched *taxon set*}| denotes the number of significantly enriched taxon sets, |{diseases}| denotes the number of diseases which is related to microbes within the same co-module. Generally speaking, higher *EI_s_* indicates good clustering quality of identified co-modules.

## Experimental Results and Discussion

### Results and Comparison

We compared MDNMF with typical NMF and NetNMF (Chen and Zhang, 2018) (without considering microbial phylogenetic information and symptoms-based disease similarity) by applying them to HMDAD dataset. Since NMF-based algorithms cannot guarantee a global optimal solution, we run 50 times with different initializations and selected the factorization with minimal objective function value as the downstream analysis.

We adopted *EI* (as described in *Functional Enrichment Analysis for Co-Modules*) and the number of significantly enriched microbe taxon set (*TS_sig_*) as metrics to evaluate the performance of different algorithms. Other taxon sets (*OTS*=|{*significantly* enriched *taxon set*}|=|*identified disease*-*related* taxon sets|) indicate the significantly enriched taxon sets that are not considered by *EI*. To some extent, the number of other taxon sets reflects the identified ability of different methods in potential microbe function modules discovering. Extensive comparison experiments are conducted and the results are shown in **Table 1**.

**Table 1:** The performance of three co-model discovering algorithms in term of *EI* and *TS_sig_*.

	(#) identified co-modules	*EI*	(#) *TS_sig_*	*OTS*
NMF	12	0.08676	39	29
NetNMF	13	0.11563	49	36
MDNMF	14	0.30182	62	48

*(P-value < 0.005 and FDR < 0.05). # represents the number of identified co-modules or significantly enriched taxon sets.

As **Table 1** shown, compared with other two NMF-based algorithms, MDNMF achieves the best performance in terms of *EI* and *TS_sig_*, indicating that MDNMF could potentially discover the meaningful function modules as much as possible by introducing symptoms-based disease network and microbe phylogenetic distance.

### Comparison of All the Significantly Enriched Taxon Sets of Modules Identified by MDNMF, NMF, and NetNMF

To demonstrate the effectiveness of MDNMF, we compared the microbe modules identified by these three approaches in terms of biologically functional enrichment. We performed microbe taxon set enrichment analysis for these three groups of modules and reserved the taxon set (*TS*) terms (FDR < 0.05, hypergeometric test) which are significantly enriched by two modules derived of MDNMF and NetNMF (or NMF). Then, for each *TS* term, we calculated enrichment scores (-log10(*p*-*value*)) and took the highest scores among all modules as the final score of this *TS* for each method. Note that the co-modules identified by MDNMF cover about 20 microbes and 3 diseases on average. There is only one co-module which contains no diseases. This is consistent with the average size of each microbe or disease module (see *Parameter Analysis*).

**Figure 2:**
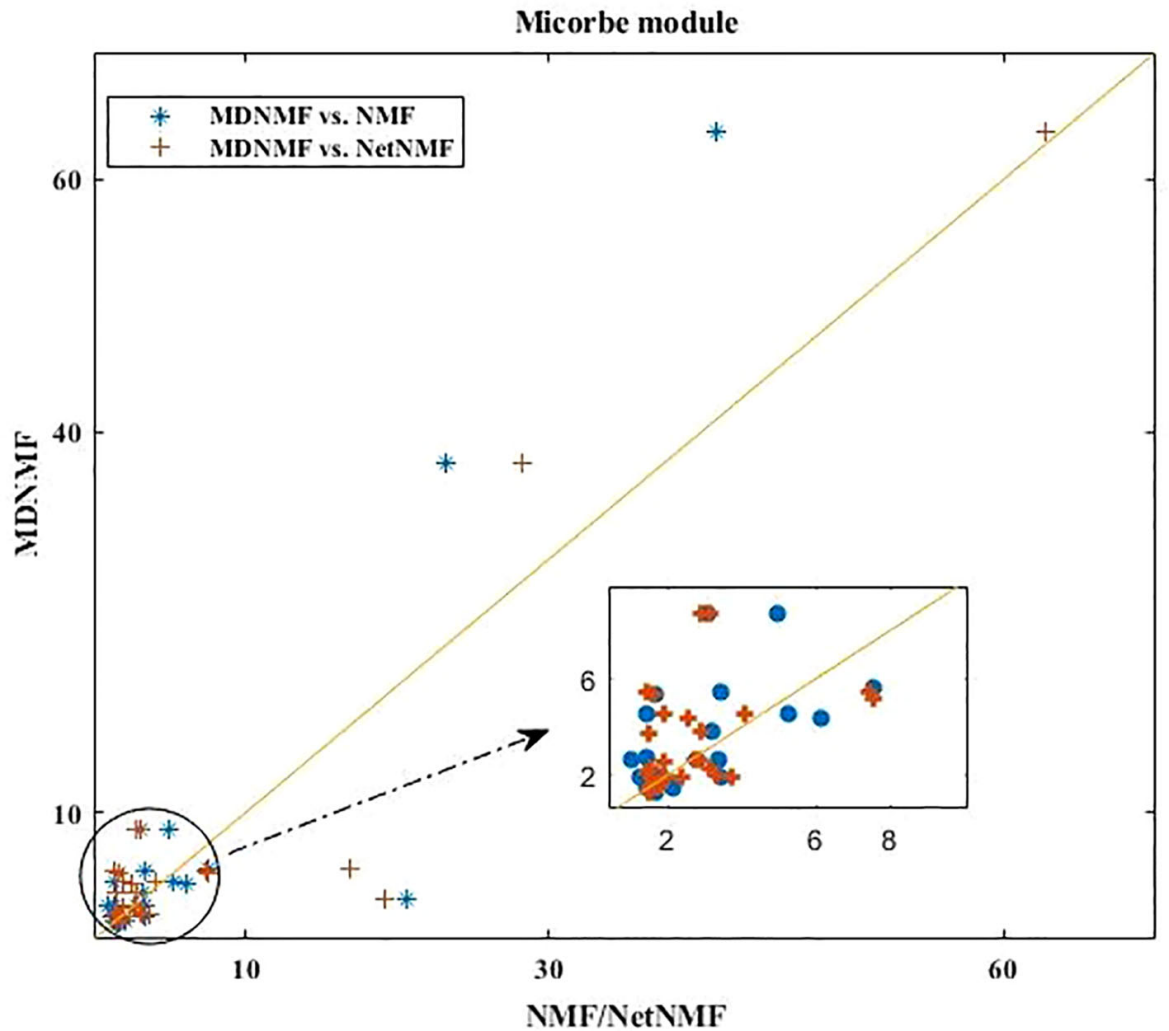
Comparison of all the enriched *TS* terms of microbe modules detected by MDNMF, NMF, and NetNMF using HMDAD dataset.

Applying MDNMF to HMDAD dataset, many *TS* terms are above the diagonal line (see **Figure 2**). Specifically, the enriched *TS* terms obtained by MDNMF have more significant Q-value (FDR < 0.05) than those of NMF and NetNMF. For microbe modules, 58.33% (MDNMF versus NMF, P < 0.005 and FDR < 0.05, hypergeometric test) and 47.06% (MDNMF versus NetNMF, P < 0.005 and FDR < 0.05, hypergeometric test) *TS* terms are above the central diagonal line, respectively.

As **Figure 2** shown, compared to NetNMF, microbe modules identified by MDNMF had lower significance for 52.94% modules. One of the possible reasons is that when selecting microbes, NetNMF just concerns the relationships among microbes from the original microbe-disease association matrix, whereas MDNMF has to take their phylogenetic relationships into account. This kind of extra constrains of MDNMF might affect the selected microbe subsets and their enriched functions. Despite that, MDNMF still identified more significantly enriched taxon sets than NetNMF (62 vs. 49, **Table 1**).

### Parameter Analysis

**Figure 3:**
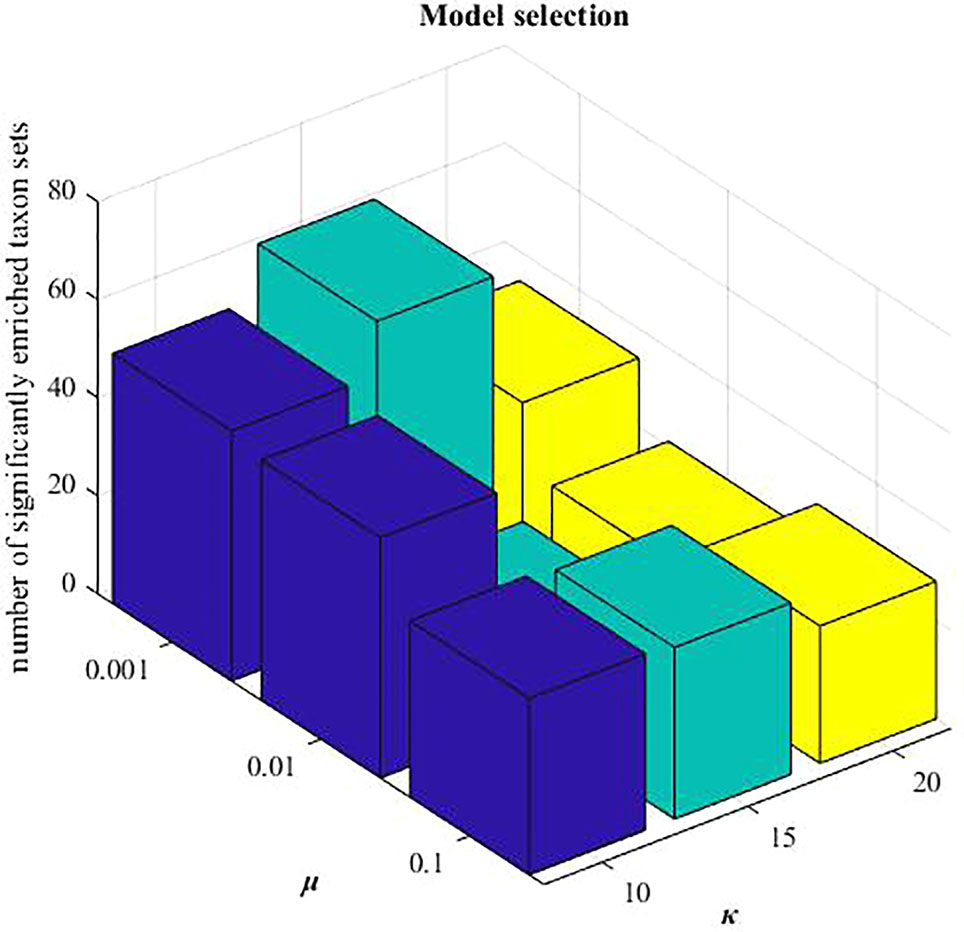
Model selection of parameters: *μ* and *k*.

In MDNMF, there are three parameters:λ_1_, λ_2_^,^ and *µ*. We set $\lambda_{1}=\frac{n_{m}}{n_{d}}$, $\lambda_{1}=\frac{n_{m}^{2}}{n_{d}^{2}}$ according to the previous study (Chen and Zhang, 2018). When applying these three NMF-based algorithms to HMDAD data, the reduced dimension *k* is needed to be pre-determined. Here, we selected *k*=15 from the candidate set {10,15,20}, and *µ*=0.001 from {0.001,0.01,0.1}, respectively. Under this setting, the number of identified microbe modules with significantly enriched taxon sets terms is highest (hypergeometric tests, P-value < 0.005 and FDR < 0.05). Mode selection is demonstrated in **Figure 3**.

### Case Studies

To further validate the performance of MDNMF, we select several microbe-disease co-modules identified by MDNMF to analyze their biological functions and inner connections. In total, 60% microbe modules are enriched in at least one *TS* term. In these identified microbe-disease co-modules, the diseases caused by microbes also exist in their matched disease modules. **Tables 2** and **3** show two of the identified microbe-disease co-modules and the associations between different disease (microbe) modules (according to *S*_2_). As *The MDNMF Algorithm* shown, in tri-factor NMF *X*≈*HS_H_^T^*, the matrix *S* has a special meaning. To see this, let us assume that *H^T^H*=*I*. Setting the derivative $\partial \min{\left\|X-HS_{H^{T}}\right\|}^{2}/\partial S$ to be 0, we can obtain:

(14) $$S=H^{T}XH,\;or\;S_{lk}=h_{l}^{T}Xh_{k}=\frac{\sum_{i\in C_{l}}\sum_{j\in C_{k}}x_{ij}}{\sqrt{n_{l}n_{k}}}.$$

**Table 2:** The identified microbe-disease co-modules by MDNMF.

Co-module_id	Disease module	Microbe module	Taxon sets (matched disease, descending order by FDR)	Associated co-module
9	*Bacterial Vaginosis* Clostridium difficile infection(CDI) *Ileal Crohn's disease(CD)* *Irritable bowel syndrome(IBS)* *Liver cirrhosis* Necrotizing Enterocolitis Periodontal *Type 1 diabetes*	*Actinobacteria* *Bacteroidaceae* *Bacteroides* *Bacteroides uniformis* *Bacteroidetes* *Firmicutes* *Fusobacteria* *Fusobacterium* *Haemophilus* *Lachnospiraceae* *Lactobacillus* *Prevotella* *Proteobacteria* *Streptococcus* *Veillonella*	*Liver Cirrhosis* Chronic Obstructive Pulmonary Disease *Bacterial Vaginosis (increase)* Asthma Colorectal Carcinoma Resistance to Immune Checkpoint Inhibitors (increase) *Type I Diabetes* *Diarrhea Irritable Bowel Syndrome (IBS)* Parkinsons (increased) Third Trimester (vs First Trimester, increase) *Crohn's Disease*	10,4,7

* Colors indicate different diseases or enriched taxon sets.

**Table 3:** The detailed information of identified microbe-disease co-module 4.

Co-module_id	Disease module	Microbe module	Taxon sets (matched disease, descending order by FDR)	Associated co-module
4	*Allergic sensitization*	*Acinetobacter* *Bacteroides ovatus* *Bacteroides vulgatus* *Burkholderia* *Clostridium coccoides* *Clostridium difficile* *Clostridium leptum* *Dietzia maris* *Escherichia coli* *Lysobacter*	*Cystic Fibrosis* *Atopic dermatitis* Aging (decrease) Dandruff *Crohn's Disease (increase)* Head and neck squamous cell carcinoma (increase)	9,7
	*Constipation IBS*			
	COPD			
	*Cystic fibrosis*			
	*Eczema*			
	*IBD*			
	New-onset untreated rheumatoid arthrits			
	*Psoriasis*			
	Rheumatoid arthrits			
	*Ulcerative colitis*			

*Colors indicate different diseases or enriched taxon sets.

*S* indicates proper normalized within-cluster sum of weights (*l* = *k*) and between-cluster sum of weights (*l* ≠ *k*). Therefore, *S* provides a good representation for the clustering quality. If the clusters are separated well, respectively the diagonal elements of *S* will be much larger than the off-diagonal elements. We conduct extensive experiments, and find that some off-diagonal elements are large, for example co-modules 4 and 9. According to Eq.14, this case may reflect a close connection between these two modules. The connections can provide some insights to further understand the relationships between microbe and disease, disease and disease, and microbe and microbe.

**Figure 4:**
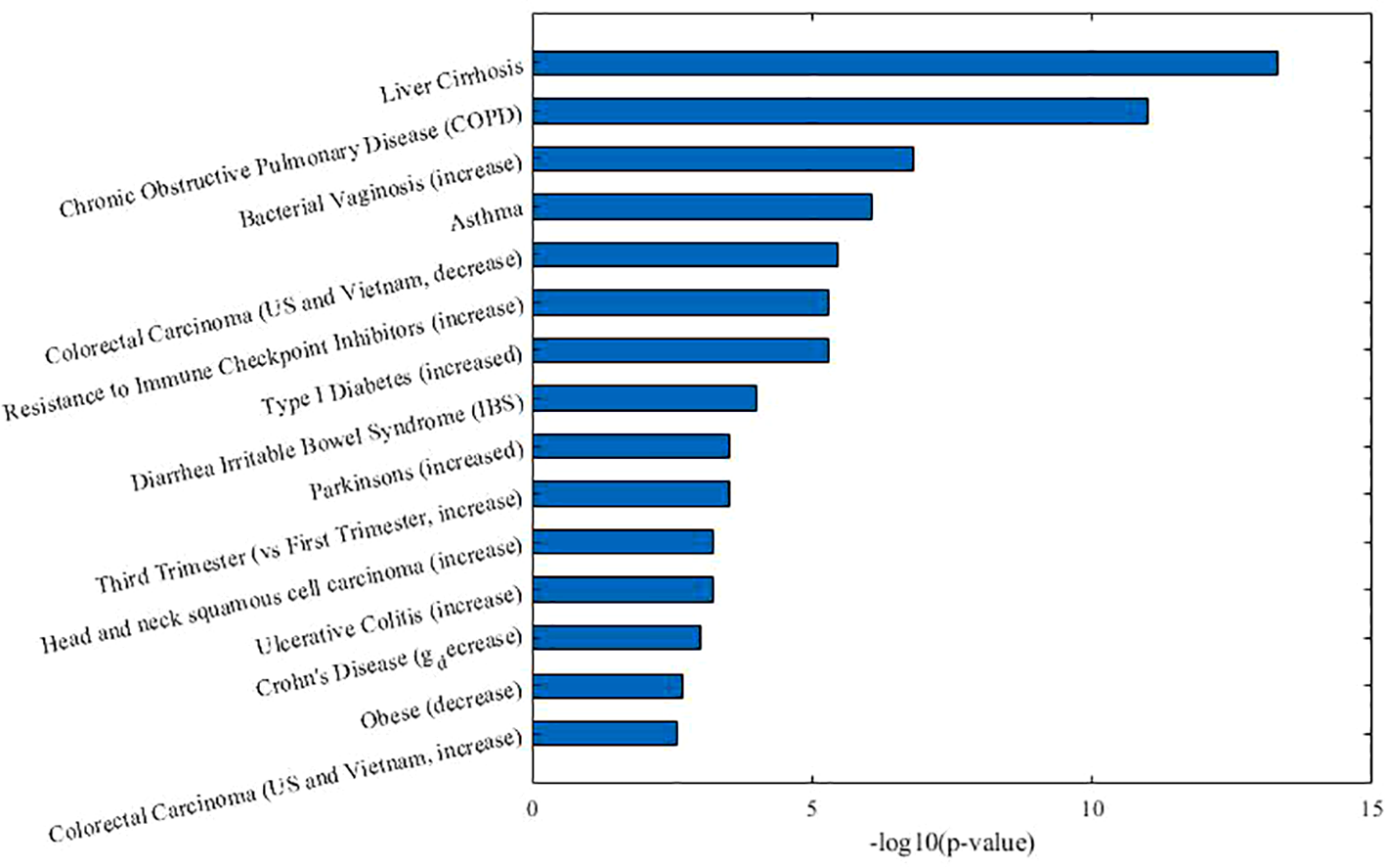
Top enriched biological terms in microbe module 9.

As **Table 2** shown, in co-module 9, 5 of 8 diseases (62.5%, same color from disease module and taxon sets columns indicates matched or associated disease) are in accord with significantly enriched microbe *TS* terms (FDR < 0.05). Besides, several *TS* such as “Chronic Obstructive Pulmonary Disease,” “Asthma,” “Colorectal Carcinoma,” “Resistance to Immune Checkpoint Inhibitors (increase)” which have no matched diseases are also identified. This could provide potential associations among diseases or microbes. **Figure 4** shows top biological terms enriched in the microbe module 9.

In order to demonstrate that MDNMF can indeed cluster similar diseases to the same co-module, we retrieval each disease existed in co-module 9 from the MeSH website (https://meshb.nlm.nih.gov) and find that most of the diseases belong to the same MeSH disease category. For example, Ileal Crohn's disease (CD), Irritable bowel syndrome (IBS), Liver cirrhosis and Necrotizing enterocolitis are clustered together and they are all divided into the same MeSH disease category C06 (Digestive System Diseases). Interestingly, Clostridium infections and Bacterial vaginosis which belong to C01 (Bacterial Infections and Mycoses) are also divided into the co-module. A detailed analysis of these related diseases may yield novel insights into the more and more widely recognized the associations between microbes and human diseases.

Based on the factorized matrix *s*_2_, we identified the connections among microbe modules 9 and 4, 7, 10. For example, microbe modules 9 and 4 share the “Crohn's Disease” and “Head and neck squamous cell carcinoma” microbe sets, but focus opposite aspects. In microbe module 9, the enriched microbe *TS* term “Crohn's Disease” is decreased, but is increased in module 4. These two microbe modules may afford us an opportunity to further investigate the complicated pathogenic mechanism in system level.

Without loss of generality, we also analyzed another microbe-disease co-module 4, the detailed information is shown in **Table 3**.

**Figure 5:**
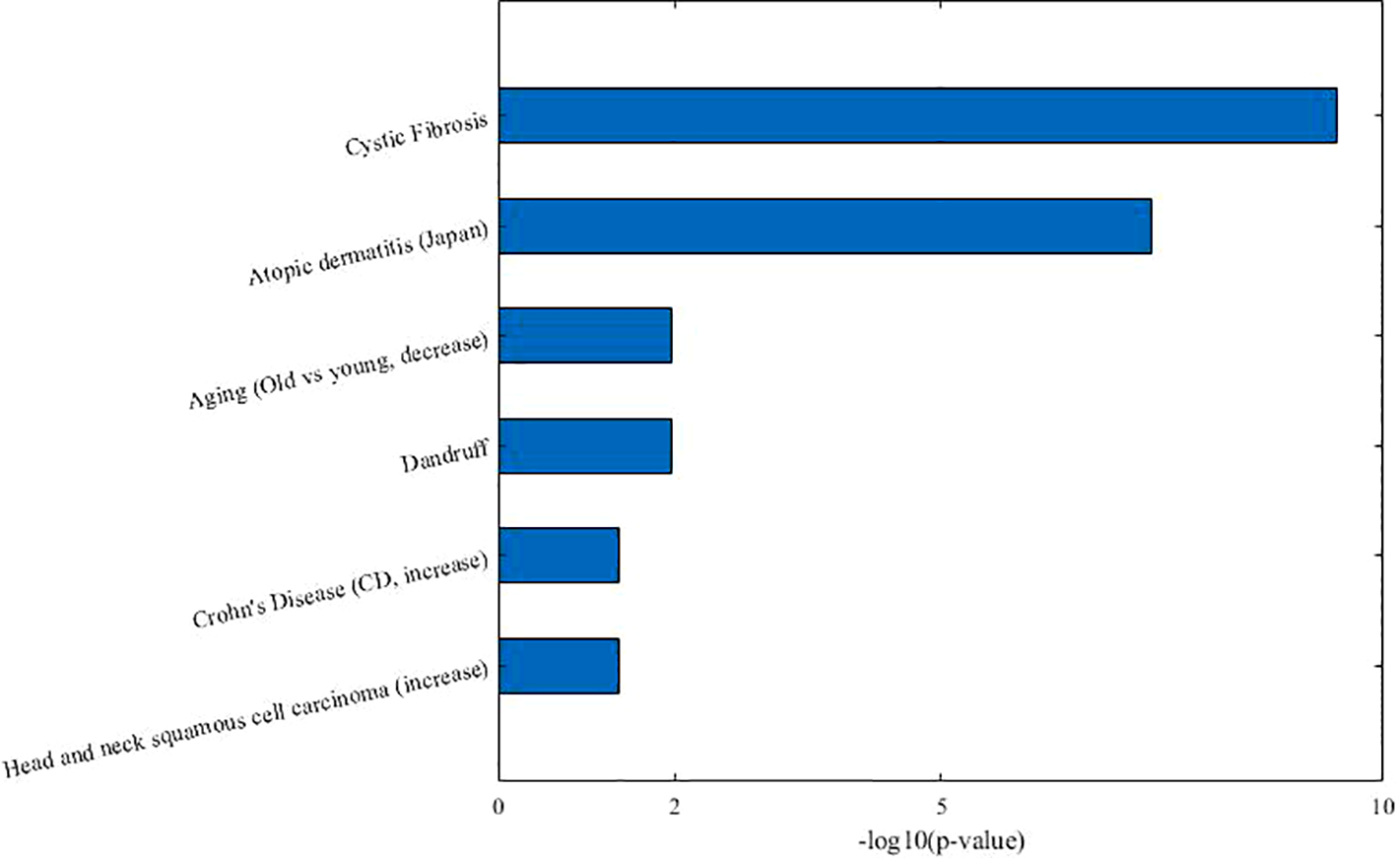
Top enriched biological terms in microbe module 4.

From **Table 3**, we can see that 7 of 10 diseases (70%, same color from the “disease module” and “taxon sets” columns indicates matched or associated disease) are in accord with significantly enriched microbe *TS* terms (FDR < 0.05). Especially, for enriched microbe *TS* term “Atopic dermatitis,” three diseases (“Allergic sensitization,” “Eczema,” and “Psoriasis”) in matched disease module are associated with it. This demonstrates the ability of the proposed MDNMF algorithm in finding correlation among diseases and microbes. **Figure 5** shows top biological terms enriched in microbe module 4.

Similarly, we retrieval each disease member in co-module 4 from the MeSH website and find that a few similar diseases belong to the same MeSH disease category. For example, Eczema, Psoriasis, Rheumatoid arthritis, and New-onset untreated rheumatoid arthritis are all from the same MeSH disease category C17 (Skin and Connective Tissue Diseases). In addition, we also find that Chronic Obstructive Pulmonary Disease (COPD), Cystic Fibrosis, Allergic sensitization, and Intestinal diseases (IBS, Irritable bowel disease, and Ulcerative colitis) have also been clustered together. Several diseases belong to two or more MeSH categories, which indicates the pathological connections between the human genetic susceptibility to infectious diseases and inflammatory diseases.

Based on factorized matrix *s*_2_, we can find that co-module 4 has more links to co-module 7(*s*_4.7_=2.72). Matched disease modules 4 and 7 own the similar disease members, such as “Allergic sensitization” (from module 4) and “Asthma” (from module 7) induced by “Atopic dermatitis.” Besides, two corresponding microbe modules 4 and 7 share *TS* term “Aging.”

Note that in **Tables 2** and **3** some related diseases and microbes are divided into different co-modules. One possible of reasons is that the connection weight between these co-modules is large, MDNMF as a soft clustering approach, cannot well separately these related microbes or disease. In the future, we will design more robust threshold selecting method to assign each diseases or microbes to accurate modules.

In summary, for the identified module pairs by MDNMF, especially for microbe modules, some of them share a few biological functions (*TS*), but also have their special roles. Simultaneously, some associations between microbe modules, disease modules can be also detected by MDNMF.

## Conclusions

The association between microbes and human diseases has been verified by more and more researches. However, previous studies mainly focused on detecting the relationship such as “one microbe, one disease,” rarely analyzed the pathogenesis of microbial-related complex diseases from a modular perspective. In this paper, we propose a novel microbe-disease co-module detecting algorithm MDNMF to construct a two-level module network by integrating two similarity matrices (microbe-microbe, disease-disease similarity matrices) and one microbe-disease bipartite network. Using the identified individual modules from different levels (microbe, disease levels) and their links, we are able to find a few disease-related microbes (taxon sets) which provide an opportunity to further understand the microbe high-order relationship and their potential functions.

Meanwhile, in order to improve the accuracy of module finding and biological interpretability of modules identified by MDNMF, we introduce human symptoms-disease network and microbial phylogenetic distance into the model. Compared with other two NMF-based approaches, MDNMF can achieve better performance in terms of *EI* and the number of significantly enriched taxon sets. The proposed MDNMF is also easily extended to other multiple-level molecular network application, for example, virus-host co-modules, microbe-drug co-modules discovering, and so on.

## Data Availability Statement

The data and MDNMF codes analyzed during the study are available in the GitHub repository, https://github.com/chonghua-1983/MDNMF.

## Author Contributions

YuM wrote the manuscript and developed the algorithms. YuM and GL developed the concept for the structure and content of the manuscript. YiM wrote the code used in the paper. QC critically revised the manuscript in final. All authors reviewed and approved the final version of the manuscript. 

## Funding

This work has been accepted by CBC2019, supported by the National Natural Science Foundation of China (No.61532008), Key Research Projects of Henan Higher Education Institutions (No. 20B520002), The Key Technology R & D Program of Henan Province (202102310561).

## Conflict of Interest

The authors declare that the research was conducted in the absence of any commercial or financial relationships that could be construed as a potential conflict of interest.
